# Interactions among insulin resistance, epigenetics, and donor sex in gene expression regulation of iPSC-derived myoblasts

**DOI:** 10.1172/JCI172333

**Published:** 2024-01-16

**Authors:** Nida Haider, C. Ronald Kahn

**Affiliations:** Section of Integrative Physiology and Metabolism, Joslin Diabetes Center, Harvard Medical School, Boston, Massachusetts, USA.

**Keywords:** Cell Biology, Metabolism, Diabetes, Insulin signaling, iPS cells

## Abstract

About 25% of people in the general population are insulin resistant, increasing the risk for type 2 diabetes (T2D) and metabolic disease. Transcriptomic analysis of induced pluripotent stem cells differentiated into myoblasts (iMyos) from insulin-resistant (I-Res) versus insulin-sensitive (I-Sen) nondiabetic individuals revealed that 306 genes increased and 271 genes decreased in expression in iMyos from I-Res donors with differences of 2-fold or more. Over 30 of the genes changed in I-Res iMyos were associated with T2D by SNPs and were functionally linked to insulin action and control of metabolism. Interestingly, we also identified more than 1,500 differences in gene expression that were dependent on the sex of the cell donor, some of which modified the insulin resistance effects. Many of these sex differences were associated with increased DNA methylation in cells from female donors and were reversed by 5-azacytidine. By contrast, the insulin sensitivity differences were not reversed and thus appear to reflect genetic or methylation-independent epigenetic effects.

## Abstract 2

Transcriptomics of iPSC-derived myoblasts demonstrates altered gene expression in insulin resistance along with a major effect of donor sex driven by epigenetic changes.

## Graphical abstract

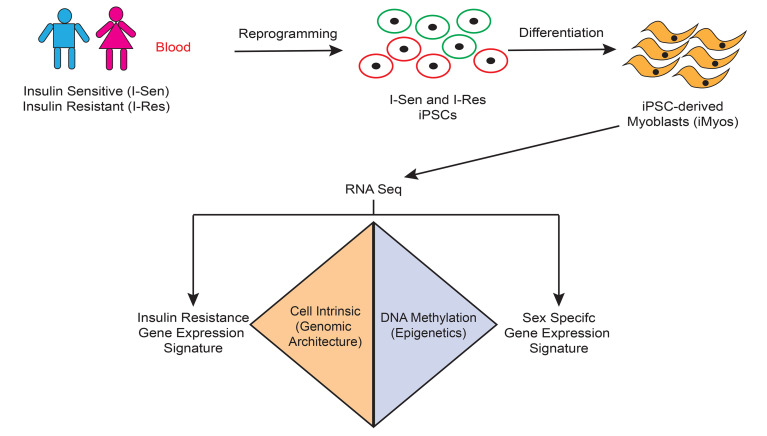

## Introduction

Insulin resistance underlies the development of type 2 diabetes (T2D) and metabolic syndrome (1–3), which together affect 20%–30% of adults in Westernized populations (4). In addition, an almost equal fraction of nondiabetic people within the general population have been shown to have a level of insulin resistance similar to that of patients with T2D when assessed by levels of circulating insulin, homeostatic model assessment for insulin resistance (HOMA-IR), euglycemic clamp, or steady-state plasma glucose in response to a fixed insulin and glucose infusion (5, 6). The molecular determinants underlying insulin resistance in these states include both cell-autonomous factors, such as genetics and epigenetics, and effects of extrinsic circulating factors, including lipids, cytokines, and miRNAs, that can modify insulin action (7). These factors likely play a role in insulin resistance in the nondiabetic population. While both T2D and metabolic syndrome affect men and women almost equally, different factors may play a role in development of insulin resistance and its metabolic complications in men and women, including differences in fat distribution and effects of circulating sex hormones (8, 9). These may lead to differences in disease progression at different stages of prediabetes (10, 11) and differences in insulin resistance–associated diseases such as atherosclerosis, fatty liver disease, and Alzheimer’s disease (12–14).

To begin to identify the cell-autonomous factors driving insulin resistance, in previous studies, we have developed a unique model using induced pluripotent stem cell–derived (iPSC-derived) myoblasts (iMyos) taken from either type 2 diabetic patients and controls (15) or insulin-sensitive (I-Sen) and insulin-resistant (I-Res) nondiabetic individuals (16) and investigated basal and insulin-stimulated protein phosphorylation using quantitative global phosphoproteomics. This revealed a broad network of cellular signaling defects associated with insulin resistance in both patients with T2D and I-Res individuals without diabetes. We also found phosphorylation differences in the iMyos taken from the male versus female individuals, especially in the cells from the nondiabetic population, reflecting possible sex-dependent differences in cellular effects (16). The goal of the current study was to investigate the determinants of insulin resistance and sex-dependent differences within the nondiabetic population by analysis of gene expression.

In the current study using RNA-Seq, we found a major effect of insulin resistance on gene expression with almost 600 up- or downregulated genes in iMyos from I-Res individuals, including many genes with SNPs linked to T2D. In addition, we observed over 1,500 differences in gene expression that were linked to the sex of the cell donor, most independent of the insulin sensitivity status, over 90% of which were on autosomal chromosomes. Furthermore, we found an increase in global DNA methylation in iMyos created from cells of female versus male individuals, and treatment with a DNA methyltransferase (DNMT) inhibitor, 5-azacytidine (5-Az), reversed some of the sex-related differences in gene expression, and a functional readout of sexual dimorphism, RhoA activation. By contrast, 5-Az did not impact the gene expression changes or differences in glucose uptake associated with insulin resistance. Thus, iPSC-derived human myoblasts exhibit differences in gene expression based on insulin resistance status and sex of the donor. The latter appear to be in large part the result of epigenetic DNA methylation changes, whereas the former appear to be mediated by a complex of genetic differences or epigenetic mediators other than DNA methylation.

## Results

### Transcriptional profiling reveals the cell-autonomous changes in gene expression associated with insulin resistance.

**Figure 1:**
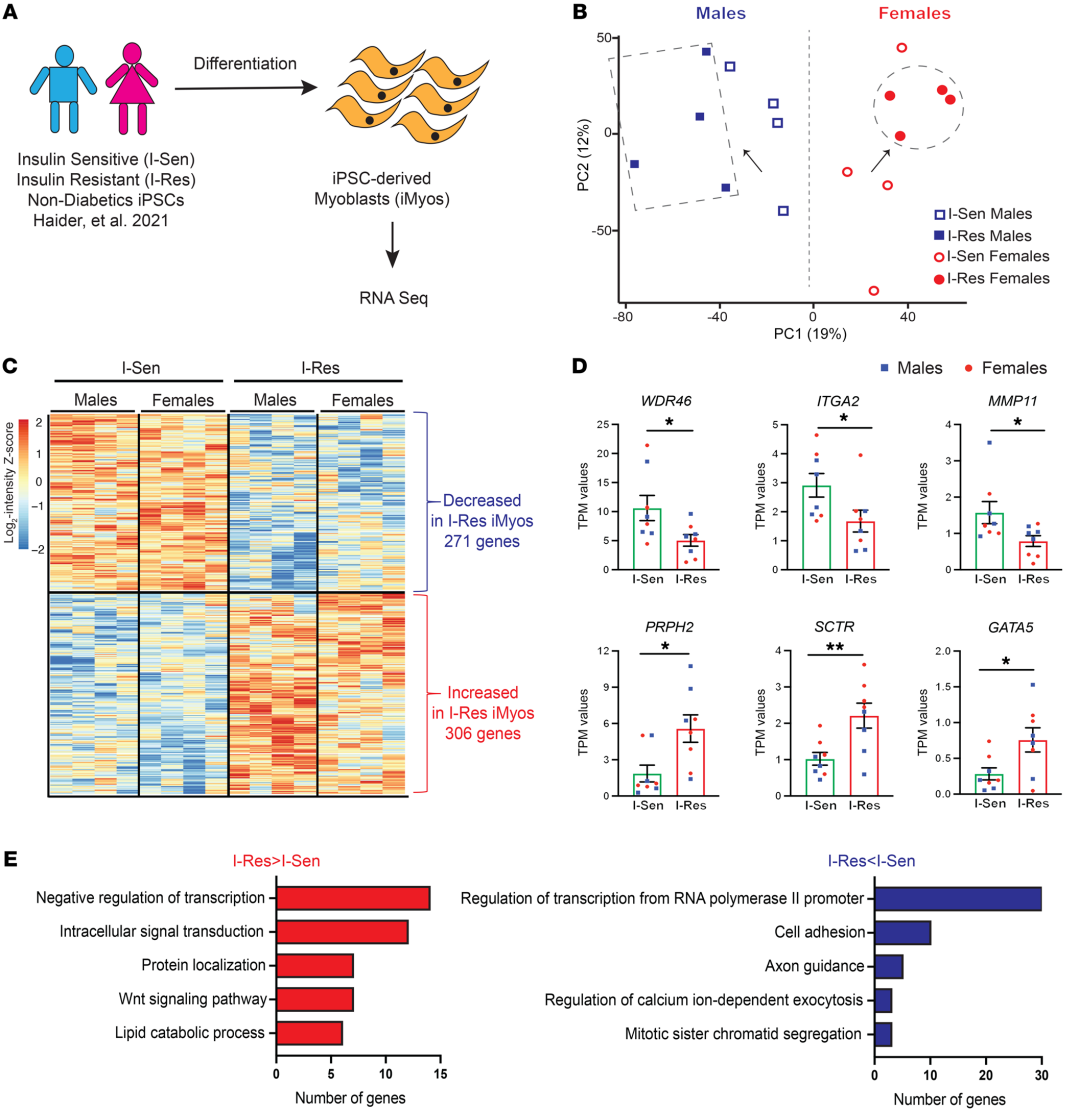
Transcriptional profiling reveals the cell-autonomous changes in gene expression associated with insulin resistance. (**A**) Schematic overview of the experimental design. (**B**) PCA plot showing the separation of the gene expression data by sex and insulin sensitivity status (open shapes, I-Sen; filled shapes, I-Res). (**C**) Hierarchical clustering of the genes showing differences associated with insulin resistance in both male and female participants. Rows represent *z* scores of the log_2_-transformed intensity of genes for each sample labeled in the column. (**D**) Quantification of representative genes from the top cluster, decreased in I-Res, and bottom cluster, increased in I-Res. Green bars, I-Sen; red bars, I-Res. Data are shown as the mean ± SEM, *n* = 8 per group (4 males and 4 females). *P < 0.05, **P < 0.01, unpaired *t* test. TPM, transcripts per million. (**E**) DAVID biological Gene Ontology (GO) analysis (*P* < 0.05) of the genes in **C** showing increased (red, I-Res > I-Sen, left) and decreased expression (blue, I-Res < I-Sen, right) in I-Res male and female iMyos.

iPSCs were created from 20 individuals without diabetes, half in the top quintile of insulin sensitivity (I-Sen) and half in the bottom quintile of insulin sensitivity, i.e., most insulin resistant (I-Res), previously identified by population screening using the steady-state plasma glucose (SSPG) approach (17); the iPSCs were derived from blood cells using nonintegrative Sendai virus (18). Both the I-Sen and I-Res cohorts were equally divided between male and female individuals and had an average age of approximately 60 years (clinical details in ref. 16 and Supplemental Figure 1; supplemental material available online with this article; https://doi.org/10.1172/JCI172333DS1). The iPSCs were converted to myoblasts (iMyos) using a 2-stage cocktail approach, and both groups of cells showed similar myogenic differentiation capacity (16). To identify the full spectrum of gene expression changes associated with the differences in insulin sensitivity, we performed RNA-Seq of iMyos from 8 I-Sen and 8 I-Res donors (Figure 1A).

Principal component analysis (PCA) of these data demonstrated a clear separation based on 2 factors: the sex of the cell donor, which was the largest driver of variance (component 1), and insulin sensitivity status, which was the second largest driver (component 2) (Figure 1B). Interestingly, in male individuals, insulin resistance status shifted the relative PCA coordinates to the left (filled squares vs. open squares, Figure 1B), whereas in female individuals, insulin resistance shifted the coordinates toward the right (filled circles vs. open circles, Figure 1B) in the PCA plot, suggesting an interaction between insulin sensitivity and donor sex at the level of gene expression. Hierarchical clustering analysis of the expression data focusing on genes that were differentially abundant between I-Sen and I-Res iMyos in both male and female individuals revealed 271 genes that were significantly decreased and 306 genes that were significantly increased in cells from I-Res donors as compared with I-Sen donors (*P* < 0.05; Figure 1C and Supplemental Table 1). The genes altered in expression in insulin resistance extended well beyond genes typically linked to insulin action but did include a number that have been previously linked to diabetes. Thus, among the decreased genes were WD repeat domain 46 (*WDR46*), which has been associated with diabetic retinopathy (19), integrin subunit α2 (*ITGA2*), which has been associated with T2D and its complications (20), and matrix metalloproteinase 11 (*MMP11*), which protects against T2D in mice (21), all of which showed 50%–70% decreases in cells from the I-Res donors (Figure 1D). Some representative examples of genes increased in expression in I-Res cells included peripherin 2 (*PRPH2*), which is associated with inherited retinal dystrophy (22), the secretin receptor (*SCTR*), which has a GWAS risk allele for development of T2D (23), and *GATA5*, a transcription factor involved in multiple processes, including pancreatic development (24), all of which exhibited 2- to 3-fold increases in cells from I-Res donors. Database for Annotation, Visualization and Integrated Discovery (DAVID) Gene Ontology analysis of the genes with decreased expression in insulin resistance revealed that the most enriched biological processes were for genes involved in the regulation of transcription (30 genes), cell adhesion (10 genes), axon guidance (5 genes), calcium ion–dependent exocytosis (3 genes), and mitotic sister chromatid segregation (3 genes) (Figure 1E, blue bars), whereas Gene Ontology analysis of genes whose expression was increased in insulin resistance identified enrichment of genes involved in negative regulation of transcription (14 genes), intracellular signal transduction (12 genes), protein localization (7 genes), Wnt signaling pathway (7 genes), and lipid catabolic process (6 genes) (Figure 1E, red bars). Mapping of the specific genes associated with each of these biological processes revealed a unique network of genes associated with insulin sensitivity status that is maintained in vitro in these differentiated iMyos (Supplemental Figure 2A). Positional gene enrichment analysis of the genes that were increased or decreased in iMyos in relationship to insulin resistance (Figure 1C) revealed that these genes are spread throughout all autosomes and the X, but not Y, chromosome (Supplemental Figure 2B). Despite its small size, there were 29 genes with differential expression on chromosome 19. Whether this represents some enrichment or simply reflects the gene-rich nature of chromosome 19, which contains roughly 1,500 genes or 6% of all the genes, remains to be determined.

Overlapping the genes that were significantly increased or decreased in I-Res iMyos (Figure 1C) with the genes associated with T2D via SNPs (19) revealed a set of 32 genes (Supplemental Figure 3), 5 of which were also associated with the most changed biological processes in I-Res iMyos (indicated by asterisks in Supplemental Figure 2A). Among the genes associated with T2D and increased expression in I-Res iMyos were the zinc finger homeobox gene *TSHZ3* and tumor suppressor *WT1*, both of which are negative regulators of transcription. By contrast, genes showing decreased expression in I-Res iMyos included positive regulators of transcription, such as *PBX2*, *ZNF213*, and *IRF2BP1*. Interestingly, the lysophosphatidic acid hydrolase *ACP6*, which is associated with T2D via SNPs (Supplemental Figure 3), was increased in expression in iMyos of I-Res donors and has also been shown to be increased in expression in skeletal muscle of individuals with a family history of T2D (25). Likewise, *TRIM63* (also known as *MURF1*), a muscle-specific E3 ubiquitin ligase, was increased in I-Res iMyos and has also been found to be increased in muscle of streptozotocin diabetic mice (26). Conversely, *FBXW7*, an F-box protein that serves as the substrate recognition component of SCF E3 ubiquitin ligase, was decreased in expression in I-Res iMyos and has been found to be decreased in muscle of the Goto-Kakizaki rat model of T2D (27). Thus, iMyos exhibit a gene expression signature associated with insulin resistance even in the absence of the influence of extrinsic factors, many of which, are similar to gene expression differences in muscle of patients with T2D.

### Sex-specific gene expression changes associated with insulin resistance.

**Figure 2:**
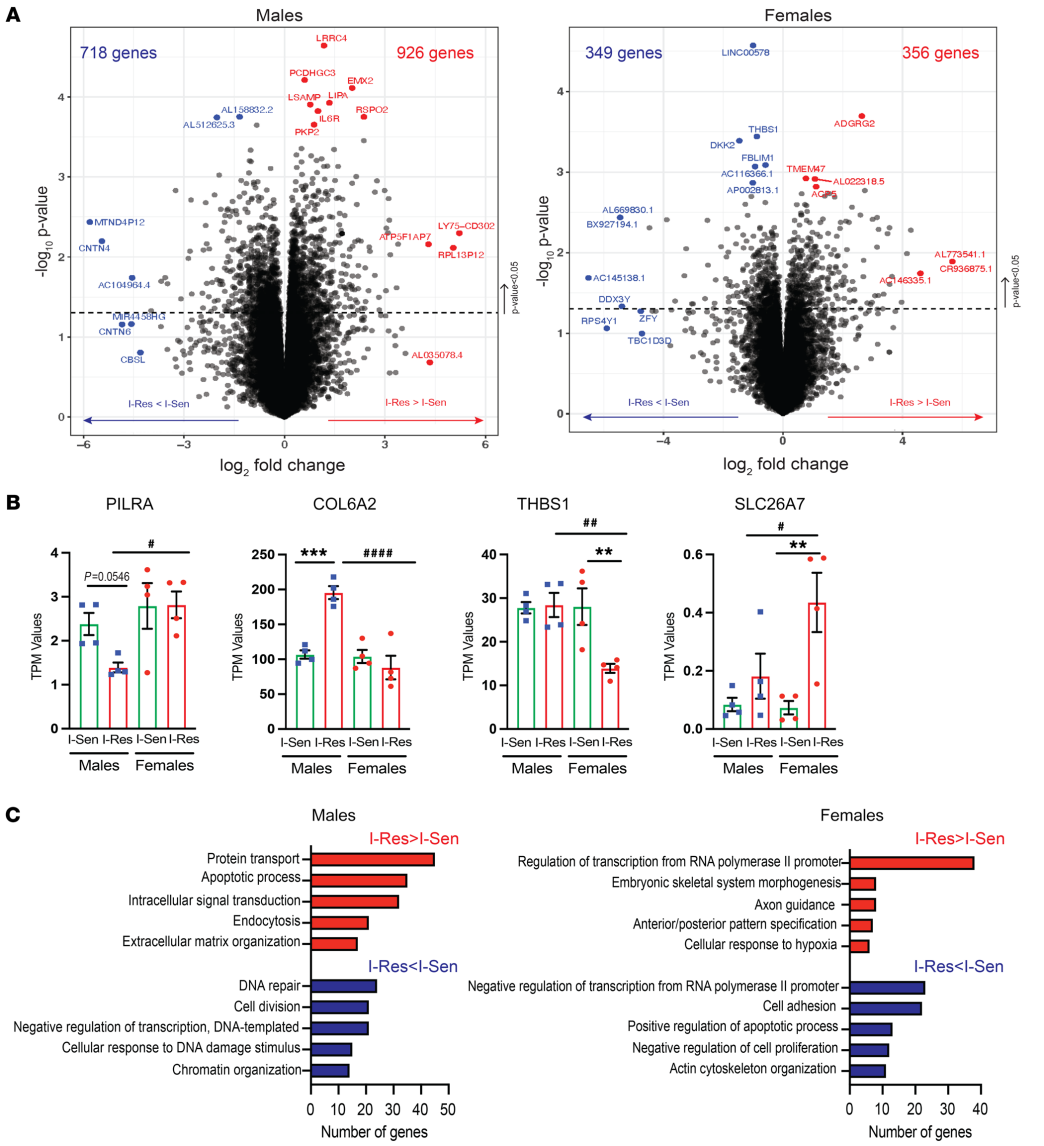
Sex-specific gene expression changes associated with insulin resistance. (**A**) Volcano plots showing gene expression increased in I-Res (I-Res > I-Sen in red) and decreased in I-Res (I-Res < I-Sen in blue) with distinct changes in male and female participants. (**B**) Quantification of representative genes showing decreased or increased levels in I-Res male (blue squares) or female (red circles) individuals. Green bars, I-Sen; red bars, I-Res. Data are shown as the mean ± SEM, *n* = 4 per group. **P < 0.01, ***P < 0.001 I-Sen vs. I-Res within one sex, or ^#^*P* < 0.05, ^##^*P* < 0.01, ^####^*P* < 0.0001 males vs. females in I-Res iMyos, 1-way ANOVA followed by correction for multiple comparison by controlling the FDR. (**C**) DAVID biological GO analysis (*P* < 0.05) of genes in volcano plots (*P* < 0.05) showing increased (red, I-Res > I-Sen) and decreased expression (blue, I-Res < I-Sen) in I-Res male (left) and female participants (right).

Because the sex of the patient has a significant effect on gene expression, we also analyzed the RNA-Seq data from iMyos of the male and female individuals separately, and this revealed an even larger set of genes impacting insulin sensitivity (Supplemental Table 2). Thus, expression of 718 genes was significantly decreased and of 926 significantly increased comparing the cells of I-Res with those of I-Sen male donors (Figure 2A, left), whereas slightly smaller numbers (349 decreased and 356 increased) were observed in the I-Res cells from the female donors (Figure 2A, right). Among the protein-coding genes that were differentially expressed, paired immunoglobulin-like type 2 receptor α (*PILRA*) showed a decrease, and collagen 6 α2 (*COL6A2*) showed a significant approximately 2-fold increase of mRNA expression in I-Res as compared with I-Sen cells from male donors but showed no changes in cells from female donors (Figure 2B). Conversely, thrombospondin 1 (*THBS1*) showed a significant 50% decrease, and solute carrier family 26 member 7 (*SLC26A7*) showed a significant approximately 5-fold increase in I-Res as compared with I-Sen cells from female individuals, with no significant changes in the cells from male individuals (Figure 2B). Interestingly, PILRA has also been found to be decreased in skeletal muscle of patients with obesity and T2D (28), and thrombospondin 1 has been linked to β-cell lipotoxicity and diabetic retinopathy (29), suggesting an important role of these sex-specific changes in diabetes pathogenesis.

DAVID Gene Ontology analysis revealed that the biological processes associated with increased expression in I-Res male individuals (Figure 2C, left, red bars) included genes involved in protein transport (45 genes), the apoptotic process (35 genes), intracellular signal transduction (32 genes), endocytosis (21 genes), and extracellular matrix (ECM) organization (17 genes), while the most enriched biological processes in cells from females were involved in regulation of transcription (38 genes), skeletal system morphogenesis (8 genes), axon guidance (8 genes), pattern specification (7 genes), and cellular response to hypoxia (6 genes) (Figure 2C, right panel, red bars). On the other hand, biological processes associated with lower gene expression in I-Res male individuals (Figure 2C, left, blue bars) included genes involved in DNA repair (24 genes), cell division (21 genes), negative regulation of transcription (21 genes), cellular response to DNA damage stimulus (15 genes), and chromatin organization (14 genes), while downregulated genes in female cells were related to negative regulation of transcription (23 genes), cell adhesion (22 genes), positive regulation of apoptosis (13 genes), negative regulation of cell proliferation (12 genes), and actin cytoskeleton organization (11 genes) (Figure 2C, right, blue bars). Thus, in addition to the 577 gene expression differences in insulin sensitivity of both male and female individuals (Figure 1C), iMyos derived from nondiabetic I-Res and I-Sen individuals showed over 2,000 changes in gene expression based on insulin sensitivity, which were distinct in male and female individuals. In addition to these protein-coding genes, bulk RNA-Seq also revealed differential expression of a few encoded long noncoding RNAs, such as AL158832.2 and AL512625.3, and a few miRNAs, including miR8075 and miR570, which were decreased in I-Res iMyos from the male donors. Further exploration of these using small RNA-Seq is warranted.

### Cell-autonomous sexual dimorphism in gene expression and their associated genomic distribution.

**Figure 3:**
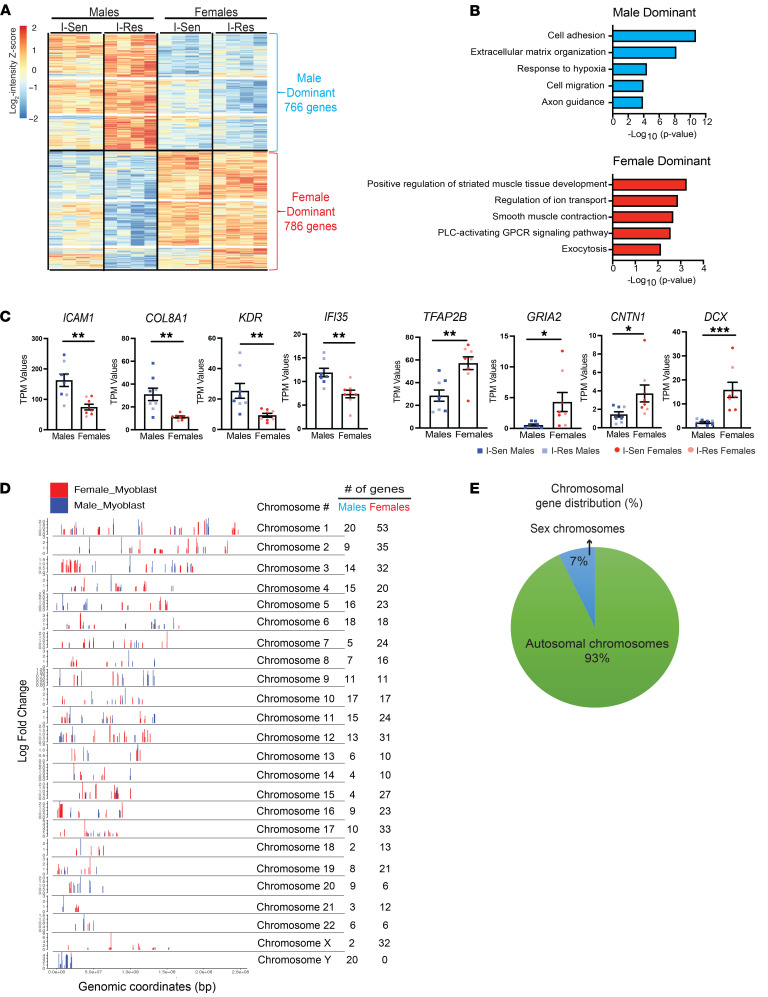
Cell-autonomous sexual dimorphism in gene expression and their associated genomic distribution. (**A**) Hierarchical clustering of the genes showing differences associated with sex of the cell donor. Rows represent *z* scores of the log_2_-transformed intensity of genes for each sample labeled in the column. (**B**) DAVID biological GO analysis (*P* < 0.05) of the genes in **A** showing male-dominant changes (blue bars, top) and female-dominant changes (red bars, bottom) in I-Res male and female iMyos. (**C**) Quantification of representative genes from the male- (blue squares) and female-dominant (red circles) clusters. Dark shade, I-Sen; light shade, I-Res. Data are shown as the mean ± SEM, *n* = 8 per group. *P < 0.05, **P < 0.01, ***P < 0.001 males vs. females, unpaired *t* test. (**D**) Positional gene enrichment analysis of 243 male-dominant and 497 female-dominant genes based on fold change >1.5 and *P* < 0.05 showing chromosomal distribution of the genes along with their genomic coordinates of the male-dominant genes (blue bars) and female-dominant genes (red bars). The height of the bar represents log fold change values. (**E**) Pie graph showing percentage of genes present on sex chromosomes and autosomal chromosomes.

Both the PCA analysis and the volcano plots in Figure 2A demonstrate that in addition to insulin resistance, the sex of the cell donor is a major modulator of differences in gene expression. Hierarchical clustering analysis of the expression data focused on sex of the cell donor rather than insulin sensitivity status revealed 1,552 genes that differed significantly in expression between male and female cells, with 766 genes being significantly higher in expression in cells from male individuals as compared with those from female individuals (i.e., male dominant) and 786 genes being significantly higher in cells from female individuals (i.e., female dominant) (Figure 3A and Supplemental Table 3).

DAVID Gene Ontology analysis revealed that the biological processes associated with the male-dominant cluster included genes involved in cell adhesion, ECM organization, response to hypoxia, cell migration, and axon guidance, while genes more highly expressed in female cells were involved in muscle tissue development, regulation of ion transport, muscle contraction, GPCR signaling, and exocytosis (Figure 3B). The magnitude of these differences ranged from 2- to 10-fold. For example, in the male-dominant cluster, *ICAM1* showed 2-fold higher levels in cells of male versus female individuals, *COL8A1* and *KDR* showed 2.8-fold differences, and *IFI35* showed a 1.7-fold difference (Figure 3C). Representative genes associated with the biological processes identified in the female-dominant clusters included *TFAP2B*, which showed a 2-fold increase, *CNTN1*, which had a 2.9-fold increase, and the glutamate receptor *GRIA2*, and doublecortin *DCX* genes, which showed 8-fold higher levels in female as compared with male cells (Figure 3C). While *DCX* is an X chromosome–encoded gene, *GRIA2* is encoded on chromosome 4 with sex differences in expression in cochlea (30) and the brain (31). These sex-specific differences were, in general, independent of the insulin sensitivity status (compare dark- vs. light-shaded squares and circles in Figure 3C) and occurred in vitro in the absence of sex hormones, i.e., represented cell-autonomous sex-specific changes in gene expression.

Since male and female cells differ in copy number of genes represented on the X and Y chromosomes, we performed positional gene enrichment analysis of the most differentially expressed male- and female-dominant genes to determine the chromosomal distribution of the sex-specific gene expression changes. Using criteria of fold change >1.5 and *P* < 0.05, we identified 243 male-dominant and 497 female-dominant genes. Analysis of these sex-biased genes mapped to their genomic coordinates is shown in Figure 3D. Importantly, 93% of the male- and female-specific genes were distributed across the autosomal chromosomes (nos. 1–22), and only 7% were localized to the sex chromosomes (X, Y) (Figure 3E). As expected, all the genes that mapped to the Y chromosome were male-dominant genes, and most of the genes mapping to the X chromosome were female dominant (Figure 3D). Most sex-differential genes that were encoded on the autosomes were widely dispersed but showed a few potential “hot spots” of activity, including a female-dominant cluster of 20 genes on chromosome 3 (genomic coordinates: 6.8 × 10^6^ to 52.8 × 10^6^) and a second female-dominant cluster of 9 genes on chromosome 16 (genomics coordinates: 0.98 × 10^6^ to 6.1 × 10^6^) (Figure 3D).

### Autosomal sex-specific gene expression changes are independent of X chromosome dosage and androgen receptor action.

**Figure 4:**
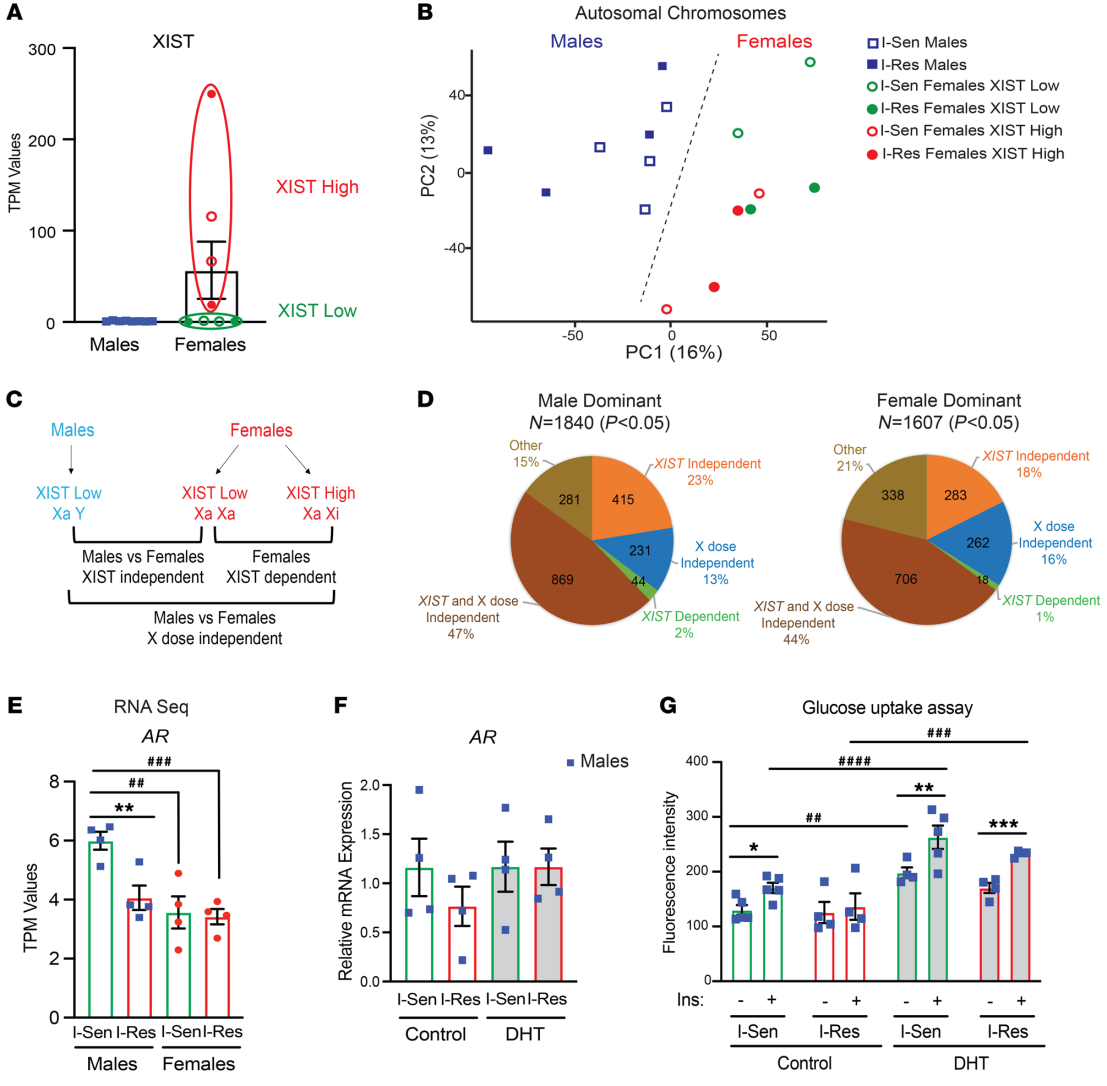
Autosomal sex-specific gene expression changes are independent of X chromosome dosage and androgen receptor action. (**A**) mRNA expression level of *XIST* in I-Sen and I-Res iMyos from male and female donors showing 2 subgroups based on *XIST* expression level, XIST high and XIST low, in female individuals. (**B**) PCA plot of only the genes present on the autosomal chromosomes showing changes based on sex (blue, males; green, XIST low females; red, XIST high females) and insulin sensitivity status (open shapes, I-Sen; filled shapes, I-Res). (**C**) Schematic overview of the data analysis comparing male individuals with XIST low and -high female individuals. (**D**) Pie graphs showing the distribution of the male-dominant genes (*n* = 1,840, *P* < 0.05) and female-dominant genes (*n* = 1,607, *P* < 0.05) based on X dose and/or *XIST* level dependency. (**E**) mRNA levels of *AR* in I-Sen and I-Res iMyos from male and female donors from RNA-Seq data. Data are shown as the mean ± SEM, *n* = 4 per group. **P < 0.01 I-Sen vs. I-Res in males, or ^##^*P* < 0.01, ^###^*P* < 0.001 I-Sen males vs. I-Sen, I-Res females, 1-way ANOVA followed by correction for multiple comparison by controlling the FDR. (**F**) mRNA levels of *AR* relative to *β-actin* in I-Sen and I-Res iMyos from male donors upon treatment with 10 μM DHT for 4 days. Data are shown as the mean ± SEM, *n* = 4 per group. (**G**) 2-NBDG glucose uptake assay in male iMyos stimulated with 100 nM of insulin for 30 minutes following pretreatment with 10 μM DHT for 4 days. Data are shown as the mean ± SEM, *n* = 4 per group. *P < 0.05, **P < 0.01, ***P < 0.001 basal vs. insulin; ^##^*P* < 0.01, ^####^*P* < 0.0001 I-Sen control vs. I-Sen DHT (basal and insulin); and ^###^*P* < 0.001 I-Res control vs. I-Res DHT (insulin); 2-way ANOVA followed by correction for multiple comparison by controlling the FDR.

Although only 7% of the sex-specific genes were localized on the X or Y chromosomes, there might be differences in X chromosome dosage in the female cells arising from the difference in the extent of X chromosome inactivation, i.e., the developmental process in which the one X chromosome in female cells is silenced by being packed into transcriptionally inactive heterochromatin (32). It is known that reprogramming of somatic cells from female donors into iPSCs results in reactivation of the silenced X chromosome, leading to 2 active X chromosomes (Xa Xa), and this is associated with a decrease in the DNA methylation of both the X chromosome and many autosomal genes (33). When iPSCs are induced to differentiate into myoblasts or other differentiated cell types, the cells undergo the process of renewed X chromosome inactivation resulting in 1 active and 1 inactive chromosome in the female cells (Xa Xi). However, this process may not be complete in all cells (34). The long noncoding RNA *XIST* is the major marker of X chromosome inactivation (35, 36). As expected, *XIST* expression was undetectable in iMyos from all the male donors (Figure 4A). By contrast, differentiated iMyos from female individuals showed variable *XIST* levels, with 4 of 8 iMyos having high levels of *XIST* expression (XIST high) and the other 4 having very low or undetectable levels of *XIST* mRNA (XIST low). This distribution was true in cells from both I-Sen and I-Res donors (Figure 4A).

To determine the effect of the sex chromosomes and different expression levels of *XIST* on the sex-specific gene expression data, we performed PCA excluding the genes encoded on the X or Y chromosome but annotated for female donors based on whether they had high or low *XIST* levels (Figure 4B). Even focusing only on autosomally encoded genes, the gene expression differences showed a clear separation based on sex of the donors (PC1 in Figure 4B), although the female donors also showed a tendency to separate by level of *XIST*, with those having low *XIST* mapping more similarly to the male donors at high values along the PC2 axis. To further explore the role of active X dosage and *XIST* expression in the changes in autosomal gene expression, we performed comparisons of the gene expression data for the XIST high (likely Xa Xi) and XIST low (likely Xa Xa) female cells versus the male cells (all XIST low) (Figure 4C). Focusing on 1,840 male-dominant and 1,607 female-dominant autosomal genes (*P* < 0.05) for these comparisons, we found that the majority of the sex-related changes in autosomal gene expression were independent of the XIST level and X chromosome dose (Figure 4D). Thus, the X chromosome dosage and the variation in *XIST* expression in female individuals do not account for most autosomal sex-specific gene expression changes.

In addition to the X chromosome dose, we also investigated the potential effect of sex hormone receptor action on the differential gene expression. Notably, estrogen receptor (*ESR1*) mRNA was not detected by either RNA-Seq or quantitative PCR (qPCR) in iMyos from either male or female donors (Figure 4E and Supplemental Figure 4A). By contrast, expression of the androgen receptor (*AR*), which, interestingly, is encoded on the X chromosome, was detected in cells of both sexes by RNA-Seq, with significantly higher levels of *AR* in the cells of I-Sen male individuals as compared with I-Res male and both I-Sen and I-Res female individuals (Figure 4E). This was confirmed by qPCR (Supplemental Figure 4A), suggesting that differences in *AR* levels in I-Sen and I-Res male individuals could potentially contribute to some of the insulin resistance changes in male individuals, as well as the sex-specific changes. Indeed, incubation of I-Sen and I-Res male iMyos with 10 μM dihydrotestosterone (DHT) for 4 days normalized the expression level of *AR* in I-Res male iMyos (Figure 4F). Interestingly, in addition, the impaired glucose uptake ability upon insulin stimulation in I-Res iMyos was also rescued upon incubation with DHT (Figure 4G). These results suggest an important role of *AR* action in regulating insulin resistance changes in male iMyos. On the other hand, overlapping the autosomal sex-specific gene expression changes in both male and female iMyos (*P* < 0.05) with RNA-Seq data of muscle from an independent study of mice with or without DHT stimulation (37) revealed that only 7.2% of the male-dominant changes and only 3% of the female-dominant changes overlapped with the DHT-induced muscle gene expression changes (Supplemental Figure 4B), suggesting that varying *AR* levels in male and female individuals do not seem to contribute to the sex-specific gene expression changes. Given that no sex hormones were added to the media used for differentiation and growth of the iMyos, and that so few of the differences in expression correspond to androgen-responsive genes, the differences in level of androgen receptor in male cells as compared with female cells do not appear to have a major impact on the expression of autosomal sex-specific genes.

### DNA methylation contributes to sexual dimorphism, but not insulin resistance.

**Figure 5:**
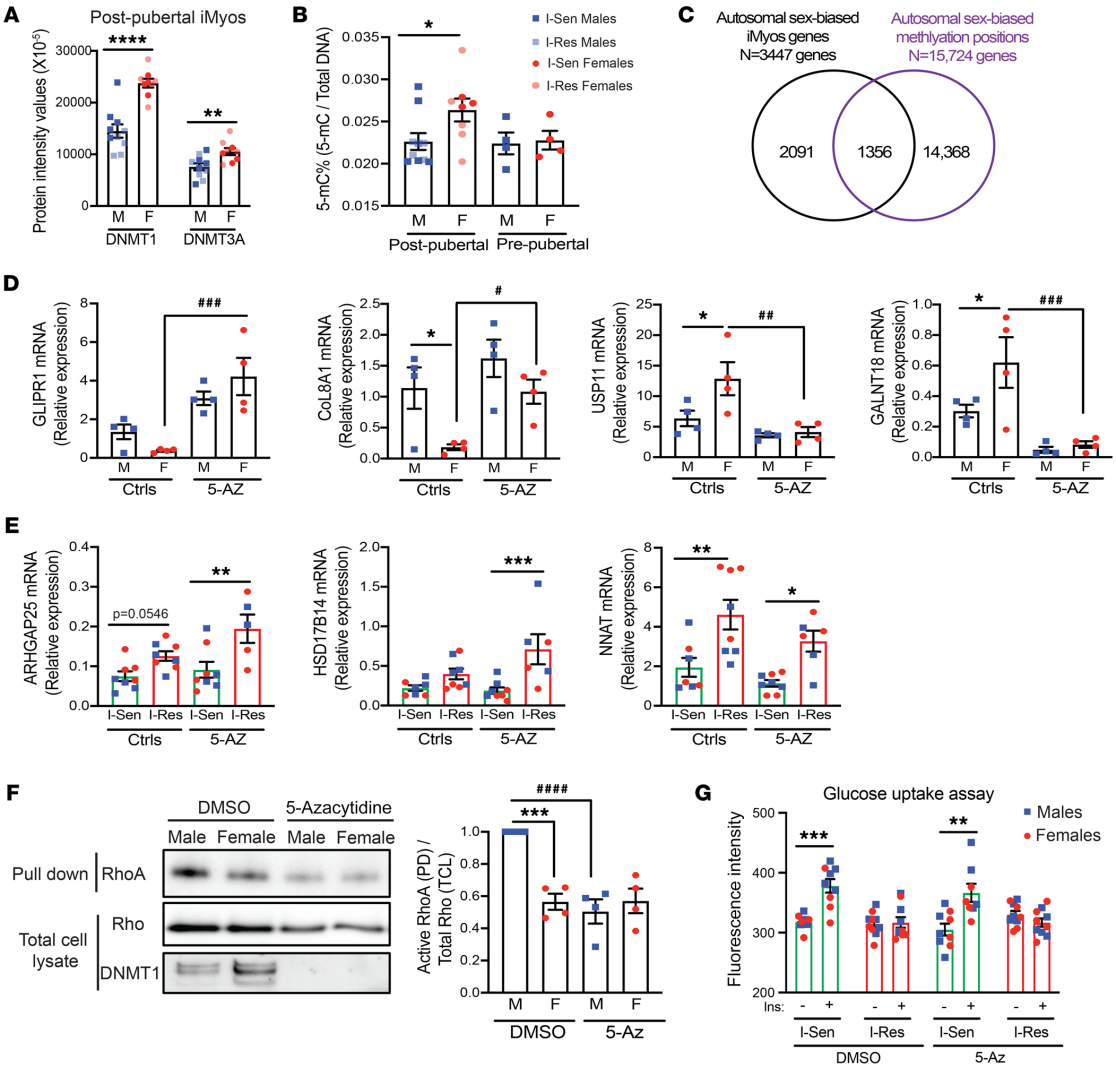
DNA methylation contributes to sexual dimorphism but not insulin resistance. (**A**) DNMT1 and DNMT3A protein levels comparing male versus female I-Sen and I-Res iMyos (blue squares, male; red circles, female; dark shade, I-Sen; light shade, I-Res) from LC-MS/MS-based proteomics. *n* = 10 males, *n* = 9 females. **P < 0.01, ****P < 0.0001 males vs. females. (**B**) ELISA measuring 5-methylcytosine (5-mC) percentage changes relative to total DNA amount in I-Sen and I-Res iMyos from postpubertal male and female donors and prepubertal male and female donors. *n* = 10 males, *n* = 8 females (postpuberty), and *n* = 4 males and females each (prepuberty). *P < 0.05 males vs. females. (**C**) Overlap of autosomal sex-biased iMyo genes (*P* < 0.05, *n* = 3,447) with autosomal sex-biased methylation positions on *n* = 15,724 genes from (from ref. 39) showing an overlap of 1,356 of 3,447 genes (39.3%). (**D**) mRNA levels of male-dominant genes, *GLIPR1* and *COL8A1*, and female-dominant genes, *USP11* and *GALNT18*, in I-Sen iMyos from male and female donors with and without 5-azacytidine (5-Az) treatment. *n* = 4 per group. *P < 0.05 males vs. females in controls, or ^#^*P* < 0.05, ^##^*P* < 0.01, ^###^*P* < 0.001 controls vs. 5-Az in females. (**E**) mRNA levels of genes altered in I-Res and I-Sen iMyos from male and female donors with and without 5-Az treatment. *n* = 5–8 per group. *P < 0.05, **P < 0.01, ***P < 0.001 I-Sen vs. I-Res. (**F**) Western blot of I-Sen iMyos from male and female cell lysates with and without 5-Az treatment processed through the active RhoA pull-down (PD) experiment and total cell lysates (TCL). Quantification of PD Western blot showing the active form of RhoA over TCL showing total RhoA levels. *n* = 4 per group. ***P < 0.001 males vs. females, ^####^*P* < 0.0001 DMSO vs. 5-Az in males. (**G**) 2-NBDG glucose uptake assay in iMyos stimulated with 100 nM of insulin for 30 minutes. *n* = 9–10 per group. **P < 0.01, ***P < 0.001 basal vs. insulin. (**A** and **B**) unpaired *t* test; (**D**–**F**) 1-way ANOVA followed by correction for multiple comparison by controlling the FDR; (**G**) 2-way ANOVA followed by correction for multiple comparison by controlling the FDR. Data are shown as the mean ± SEM.

The I-Sen and I-Res iPSCs were originally derived from circulating blood cells of adult men and women with an average age of 60 years, i.e., all donors were postpubertal, and all or most of the women postmenopausal. Thus, the donor had been exposed for many years to varying levels of sex hormones prior to cellular isolation for iPSC derivation. Although reprogramming of blood cells into iPSCs is known to erase most of the epigenetic marks exerted by hormonal action and other factors in vivo (38), it is possible that some residual epigenetic marks remain and contribute to the differences observed in the iMyos. To investigate this possibility, we assessed expression differences for some of the genes involved in epigenetic regulation in the I-Sen and I-Res iMyos from male and female donors. Interestingly, the expression of the major DNA methyltransferase *DNMT1* encoded on chromosome 19, as well as of the histone-lysine *N*-methyltransferase (*EZH1*) encoded on chromosome 17, was significantly higher in the cells from female donors as compared with those from male donors, independent of the insulin sensitivity status (Supplemental Figure 5A). These differences in gene expression were even magnified at the protein level, with 39% and 35% increases in protein expression of DNMT1 and DNMT3A (*P* < 0.0001 and *P* < 0.007), respectively, in cells from female donors as compared with those from male donors, independent of insulin sensitivity status (Figure 5A). Consistent with the difference in the expression of the methylation enzymes in the postpubertal iMyos, we found significantly high levels (~15% increased, *P* < 0.05) of global DNA methylation in cells from the postpubertal female individuals as compared with the postpubertal male individuals (Figure 5B).

To determine the potential role of postpubertal sex hormones in these epigenetic effects, we used an independent set of iPSCs derived from blood cells of normal prepubertal, i.e., less than 10 years old, male and female donors and differentiated these into iMyos. Interestingly, in the prepubertal iMyos, no differences were observed in the mRNA level of *DNMT1* and *EZH1* between the sexes (Supplemental Figure 5A). Likewise, no difference was observed in global DNA methylation in the iMyos from prepubertal donors (Figure 5B), suggesting that sex hormone exposure in vivo may result in persistent DNA methylation epigenetic marks and contribute to some of the gene expression differences observed in the cells derived from the postpubertal donors. These findings are supported by a recent study using human skeletal samples from 222 male and 147 female individuals, which revealed that, of the differentially methylated regions, 94% were hypomethylated in male participants as compared with female participants (39). Overlapping the male and female differences on autosomal genes in iMyos (*n* = 3,447 genes, *P* < 0.05) with this analysis of autosomal sex-biased methylation in human muscle samples (*n* = 15,724 genes, *P* < 0.05) revealed 1,356 genes in iMyos (39%) that overlapped with the genes showing sex-biased methylation in human muscle samples (Figure 5C). Thus, a significant proportion of the genes with sex-biased expression observed in iMyos contain differentially methylated positions.

To further investigate whether these differences in DNA methylation play a role in the sex or insulin resistance differences in gene expression, we treated postpubertal male and female iMyos with 5-azacytidine (5-Az), an inhibitor of DNMT (Supplemental Figure 5B). This did not affect cell density as assessed by crystal violet staining or protein content (Supplemental Figure 5B). mRNA expression of 2 normally male-dominant genes, glioma pathogenesis–related protein 1 (*GLIPR1*) and collagen type VIII α1 chain (*COL8A1*), showed a significant rescue of the gene expression in the female iMyos following treatment with 5-Az (Figure 5D). Similarly, mRNA expression of two female-dominant genes, ubiquitin-specific peptidase 11 (*USP11*) and *N*-acetylgalactosaminyltransferase 18 (*GALNT18*), showed a significant reversal of the increased expression in the female iMyos following treatment with 5-Az (Figure 5D), suggesting that epigenetically mediated DNA methylation contributes to these sex-specific gene expression differences. On the other hand, mRNA expression of genes increased in I-Res male and female iMyos, including Rho GTPase activating protein 25 (*ARHGAP25*), 17-β-hydroxysteroid dehydrogenase (*HSD17B14*), and neuronatin (*NNAT*), remained unaffected following treatment with 5-Az (Figure 5E), suggesting that DNA methylation is not a major contributor to the gene expression changes associated with insulin resistance.

Using phosphoproteomics, we previously showed that iMyos exhibit multiple sex-specific differences in a broad network of protein phosphorylations, several of which were related to the Rho GTPase pathway (16), leading to enhanced activation of RhoA in iMyos from males versus female donors, as measured in a pull-down assay (16). This increase in Rho GTPase activity correlated with significantly higher levels of RhoA mRNA in postpubertal male iMyos as compared with the female iMyos (Supplemental Figure 5C). We therefore used RhoA activation as a functional readout to study the impact of DNMT inhibition on sexual dimorphic functional changes. Again, we found that there were higher levels of RhoA activation in the male versus female cells from I-Sen iMyos, and this was abolished by treatment of the cells with 5-Az (Figure 5F and Supplemental Figure 5D), indicating that a methylation-dependent epigenetic modification was contributing to this sexually dimorphic functional difference. We also showed that the higher level of DNMT1 protein in the cells from female individuals as compared with those from male individuals observed by Western blotting was lost after blockade of DNMT1 upon 5-Az treatment (Figure 5F). Thus, treatment with 5-Az was sufficient to reverse the sex-biased changes in a functional readout of RhoA activation.

This reversal of a sexually dimorphic phenotype was not observed for insulin resistance as measured by glucose uptake. Thus, when we assessed glucose uptake of the I-Sen and I-Res iMyos with and without treatment with 5-Az using 2-(*N*-(7-nitrobenz-2-oxa-1,3-diazol-4-yl)amino)-2-deoxyglucose (2-NBDG) fluorescent glucose, we found that the ability of insulin to stimulate increased glucose uptake in I-Sen iMyos was markedly reduced in the I-Res iMyos, and this effect was not reversed by treatment with 5-Az (Figure 5G). Thus, while DNA methylation contributes to sexual dimorphism in gene expression and Rho activation, it does not appear to contribute to differences in gene expression related to insulin resistance or to the reduction in insulin-stimulated glucose uptake.

## Discussion

Insulin resistance is central to the pathophysiology of T2D, obesity, and metabolic syndrome. Insulin resistance can be identified in offspring of T2D parents many years prior to the onset of the disease and predicts disease development (3). In addition, insulin resistance is present in a substantial fraction of the general population, making these individuals at higher risk for the development of T2D and metabolic syndrome (6). One powerful approach to determining the cellular components of disease pathogenesis is the use of induced pluripotent stem cells (iPSCs) (40–42). iPSCs can be maintained in culture indefinitely and differentiated into almost any tissue of interest in the absence of circulating modifiers. Recently, using myoblasts generated from iPSCs (iMyos) of individuals over the range of insulin sensitivity, including patients with insulin receptor mutations (43), patients with T2D (15), and individuals without diabetes with insulin resistance (16), we found large differences in the phosphoproteome based on insulin resistance status of the donor. Many of the alterations in signaling in the iMyos of the I-Res individuals without diabetes overlap with the alterations observed in cells from patients with T2D, highlighting key steps through which to target insulin resistance (15, 16). In addition, we found that the sex of the cell donor further modifies intracellular signaling and that these changes can be reflected in differences in downstream biological responses (16). Consistent with this, sex-specific differences in insulin sensitivity in humans have also been observed in clinical studies. Hyperinsulinemic-euglycemic clamp studies have found that healthy women are more insulin sensitive than men owing to enhanced glucose uptake and a higher proportion of type I muscle fibers in women (44, 45). The goal of the current study was to determine how differences in gene expression might be associated with insulin resistance and sex-dependent alterations and contribute to these functional changes.

Using RNA-Seq, we have identified 577 genes that are altered in their expression levels in insulin resistance in cells of both male and female donors, with 306 genes increased and 271 genes decreased in I-Res versus I-Sen iMyos. Many of these form complementary networks. For example, genes related to negative regulation of transcription are increased in insulin resistance, while those related to positive regulation of transcription are decreased, suggesting an overall decrease in transcriptional activity as a component of cell-intrinsic insulin resistance. Interestingly, among this group of genes, a subset of genes have been associated with T2D through SNPs (19), including *TSHZ3* and *WT1*, which are increased in expression in I-Res iMyos, and *PBX2*, *ZNF213*, and *IRF2BP1*, which are decreased in expression in I-Res iMyos. In addition to these transcriptional regulators, 27 other genes with differential expression in I-Res iMyos have been associated with T2D through SNPs and are also functionally linked to insulin action and control of metabolism. Thus, we found increased expression of genes in I-Res iMyos for biological processes related to protein localization, Wnt signaling, lipid catabolic processes, and intracellular signal transduction, including *TGFBP3*, a gene associated with adipose biology and several inflammatory diseases (46, 47). In contrast, we found decreased expression of genes associated with cell adhesion (such as *ITGA2*), axon guidance (including *WNT3*), and calcium ion–dependent exocytosis, as well as *TONSL*, a negative regulator of NF-κB–mediated transcription, all of which have been linked to T2D through SNPs (48). Taken together, these cell-autonomous defects in gene expression associated with insulin resistance include potential for multisite transcriptional dysregulation and increased proinflammatory intracellular signaling. This is in agreement with our phosphoproteomics analysis using the same cellular model (16). Overlapping of the insulin sensitivity gene expression changes in iMyos with changes in gene expression observed in primary cultured myotubes from T2D/obese patients (49) showed an approximately 20% overlap in these gene signatures.

Superimposed on the differences related to insulin resistance, the sex of the cell donor is a major modulator of differences in gene expression. Normal development, anthropometric traits, and disease phenotypes, such as prevalence, progression, and age of onset, have all been shown to exhibit sex-differentiated characteristics. These sex-based differences are often attributed to hormones, sex chromosomes, and environmental differences, but the full extent of these differences and their underlying molecular mechanism largely remain unknown. Indeed, a recent study of gene expression in 44 human tissues revealed that 37% of all genes showed sex-biased expression differences in at least one tissue (50). To what extent these effects were created by the hormonal milieu in vivo or were intrinsic, based on the sex of the person from whom the tissues were derived, is unclear. Here, in this ex vivo system free of added sex hormones, we found over 1,500 sex-biased genes, which are independent of the insulin sensitivity status. Only about 7% of these sex-specific gene expression differences occur in genes on the X or Y chromosome, i.e., 93% of the sex-differentially expressed genes are on autosomes. Interestingly, we identified hot spots of differential expression where multiple female-dominant genes formed clusters on chromosome 3 (20 genes) and chromosome 16 (9 genes). It is possible that these hot spots correspond to specific transcription factor binding sites, methylation sites, or interaction with long noncoding RNAs (lncRNAs) leading to downstream regulation and sex-biased gene expression.

The lncRNA *XIST* has been shown to not only regulate gene expression on the X chromosome but also transregulate gene expression in some genes on autosomal chromosomes (51, 52). In iMyos, all male cell lines have undetectable *XIST* levels, whereas in cells derived from female individuals, about half have high levels of *XIST* and half have very low or undetectable levels. Analysis of the data considering the varying *XIST* expression level in the female iMyos shows that most of the sex-biased differences in gene expression are independent of the level of *XIST* expression, supporting the notion that sex chromosome dosage does not play an important role in most of these sex-specific gene expression differences. Additional studies using iPSCs from patients with Turner syndrome (XO females) (53) and/or trisomy X (XXX females) (54) might help to further define the role of X chromosome dosage in these sex-specific gene expression changes.

The iPSCs used for the development of the iMyos were derived from circulating blood cells taken from postpubertal adults, who had, therefore, been exposed to circulating sex hormones in vivo. However, in vitro, neither the maintenance nor the differentiation of the iPSCs involves addition of sex hormones, limiting the potential impact of hormones on these sex-specific gene expression changes. While this is a limitation of this cellular system, one of its strengths is its ability to assess cellular function in the absence of these extrinsic circulating factors, since our aim is to investigate the cell-intrinsic changes in insulin sensitivity in muscle. Interestingly, there are differences in sex hormone receptors in iMyos. While the estrogen receptor mRNA was not detected in the iMyos of either sex, the level of androgen receptor (*AR*), which coincidentally is encoded on the X chromosome, was higher in cells from I-Sen male individuals compared with both I-Res male and all female individuals. Testosterone deficiency in men has been associated with the development of obesity, insulin resistance, and T2D, in addition to its effects on erectile dysfunction (55). Likewise, men with prostate carcinoma receiving androgen deprivation therapy show a higher risk of developing insulin resistance and hyperglycemia (56), consistent with our findings of reduced AR expression level in I-Res iMyos. Indeed, normalizing AR levels in I-Res male individuals upon incubation with DHT rescues, at least in part, the impaired insulin-stimulated glucose uptake defect. In this perspective, clinical studies have shown that testosterone can promote insulin sensitivity in hypogonadal men with and without diabetes (57), and in women, androgen excess promotes insulin resistance (58, 59). Thus, differences in *AR* levels could account for insulin sensitivity changes in gene expression in male iMyos; however, this is not likely to be the major driver of the sex-specific gene expression changes. Indeed, known androgen-responsive genes in the muscle (37) show minimal overlap with the sex differences in gene expression in iMyos. Likewise, in ongoing work, we find that blocking AR action in iMyos by treatment with the AR antagonist bicalutamide (60) has little impact on the sex-based differences in gene expression.

In normal development, exposure to sex hormones during different developmental stages of life is known to exert epigenetic changes that can persist throughout life. Many of these are related to DNA methylation (reviewed in ref. 61). Similarly, alterations of the DNA methylation can contribute to differences in gene expression and provide a link between the development of metabolic diseases, genes, and environment (61, 62). Indeed, altered DNA methylation of genes such as *PDK4* and *PPARGC1A* has been found in skeletal muscle from patients with T2D (63–65). Here, we find significantly higher global DNA methylation in female iMyos as compared with male iMyos, independent of insulin sensitivity status, consistent with other studies, which have shown that in addition to methylation on the inactive X chromosome, female cells also show higher levels of autosomal methylation in muscle (39). Interestingly, this difference in global levels of DNA methylation was not observed in iMyos differentiated from iPSCs of prepubertal male and female individuals, suggesting that the sex hormone exposure, or other possible mechanisms such as aging, in the postpubertal female might lead to epigenetically mediated DNA methylation changes, some of which persist or reoccur through the reprogramming and differentiation process. Indeed, Landen et al. found that human skeletal muscle samples from female individuals had an increased number of differentially methylated regions as compared with muscle from male individuals (39). Overlapping our data with their data revealed that 39% of the genes exhibiting sex-differential expression in our data contain differentially methylated positions; however, 61% do not. Therefore, while DNA methylation may contribute to a significant fraction of the sex-specific differences in gene expression, the molecular mechanism underlying the majority of these genes involves mechanisms other than DNA methylation. Nonetheless, blocking DNA methylation with the DNMT inhibitor 5-Az reverses at least some of the sex-dependent differences in expression of male- and female-dominant genes, as well as a sexually dimorphic functional difference in RhoA activation.

Despite a role in sex-specific changes, DNA methylation does not appear to be the major driving force in gene expression differences related to insulin resistance or the reduced ability of insulin to stimulate glucose uptake in I-Res cells, since neither of these were changed by 5-Az treatment. This leaves open the important question of how these insulin resistance–related changes in gene expression and glucose uptake are mediated. They could be mediated by underlying genetic effects that lead to the altered transcriptional regulation observed in these cells. In addition, there could be epigenetically mediated mechanisms involving histone modification and effects of noncoding RNAs. Histones can undergo modifications involving acetylation, methylation, phosphorylation, and ubiquitination, all of which can lead to changes in chromatin structure and alterations in gene expression (66). Histone modifiers and chromatin remodelers play an important role in iPSC reprogramming and differentiation (reviewed in ref. 67), but how these are regulated in redifferentiation of iPSCs is less well studied. In addition, both long noncoding and short noncoding miRNAs can modify transcription and translation (68). Indeed, *XIST* is an important example of how a single lncRNA can affect expression of multiple genes. The role of these other epigenetic mechanisms in insulin resistance and sex-specific gene expression changes remains to be investigated.

In summary, human iPSC-derived myoblasts demonstrate a cell-intrinsic gene expression signature associated with insulin resistance. These gene expression changes are retained in cells after the reprogramming process and differentiation of iPSCs into myoblasts and appear to be independent of DNA methylation, indicating the cell-autonomous nature of these insulin sensitivity differences. Determining the mechanisms underlying these differences should provide new targets for defying insulin resistance and preventing its metabolic consequences. In addition, iPSC-derived myoblasts exhibit a large panel of differences in gene expression that are sex specific, most of which involve genes encoded by autosomal chromosomes. At least one mechanism linked to this sexual dimorphism in gene expression is differences in DNA methylation, possibly related to sex hormone exposure and epigenetic programming in the donor in vivo that either persists through iPSC reprogramming or is reintroduced during differentiation of the iPSCs into myoblasts. Understanding the impact of sex on gene expression will be important not only in insulin resistance, but also in normal physiology and pathophysiology of many diseases.

## Methods

### Study participants, SSPG, and iPSC reprogramming.

The iPSC lines were generated from 20 human study participants who had been recruited and assessed for insulin sensitivity using steady-state plasma glucose (SSPG) obtained from the modified insulin suppression test at the Stanford Clinical and Translational Research Unit (69). iPSC lines were generated as described previously (18), and those used in the study were chosen from 8 in the upper quintile of insulin sensitivity and 8 in the lowest quintile of insulin sensitivity, matched for age, sex, and race/ethnicity based on the SSPG as previously described (16).

### iPSC culture, myogenic differentiation, and 5-Az treatment.

The iPSCs were cultured on plates coated with hESC-qualified Matrigel (Corning) using the mTeSR1 medium containing the 5× complement (Stemcell Technologies) and passaged as aggregates using ReLeSR (Stemcell Technologies). For differentiation into myoblasts, a modified version of the 2-step differentiation protocol was used (70). For this, approximately 7 × 10^3^ iPSCs/cm^2^ were seeded onto collagen I–coated plates (Biocoat, Fisher) in skeletal muscle cell growth basal medium (Lonza) containing 5% horse serum, 50 μg/mL fetuin, 3 μM CHIR99021, 2 μM Alk5 inhibitor, 1 ng/mL bFGF, 10 ng/mL human recombinant epidermal growth factor (hrEGF), 10 μg/mL insulin, 0.4 μg/mL dexamethasone, 10 μM Y27632 (Rock inhibitor), and 200 μM ascorbic acid with change of medium every 2 days, which resulted in myogenic precursor/satellite-like (SC-like) cells within 10 days. The SC-like cells were then trypsinized and plated at approximately 7 × 10^3^ iPSCs/cm^2^ onto collagen I–coated plates (Biocoat, Fisher) in skeletal muscle cell growth basal medium (Lonza) containing 5% horse serum, 50 μg/mL fetuin, 10 μg/mL insulin, 0.4 μg/mL dexamethasone, 10 μM Y27632 (Rock inhibitor), 10 ng/mL hrEGF, 20 ng/mL human recombinant hepatocyte growth factor (HGF), 10 ng/mL human recombinant platelet-derived growth factor (PDGF-AB), 10 ng/mL oncostatin M, 20 ng/mL bFGF, 10 ng/mL insulin-like growth factor 1 (IGF-1), 2 μM SB431542, and 200 μM ascorbic acid with change of medium every 2 days. This resulted in well-differentiated myoblasts (iMyos) within another 10 days as characterized by high levels of MyoD1 (16).

For the treatment with 5-Az, the differentiated iMyos were treated with 20 μM 5-Az for 24 hours followed by processing for RNA extraction as described below.

### RNA isolation, qPCR, and RNA-Seq.

Total RNA from all the cell types was isolated using TRIzol (Thermo Fisher Scientific) following the chloroform/isopropanol/ethanol extraction method. Complementary DNA (cDNA) was synthesized from 400 ng of RNA using a High Capacity cDNA Reverse Transcription kit (Applied Biosystems), and the resulting cDNA was used for the qPCR reaction with iQ SybrGreen Supermix (Bio-Rad, catalog 1708884) performed on a C1000 Thermal Cycler (Bio-Rad, catalog CFX384). TATA box binding protein (Tbp) was used as a housekeeping gene to normalize gene expression unless stated otherwise. Primer sequences used were *TBP* (forward: CCACTCACAGACTCTCACAAC; reverse: CTGCGGTACAATCCCAGAACT), *AR* (forward: GACGACCAGATGGCTGTCATT; reverse: GGGCGAAGTAGAGCATCCT), *ESR1* (forward: GAAAGGTGGGATACGAAAAGACC; reverse: GCTGTTCTTCTTAGAGCGTTTGA), *DNMT1* (forward: AGGCGGCTCAAAGATTTGGAA; reverse: GCAGAAATTCGTGCAAGAGATTC), *EZH1* (forward: GTCACTGAACACAGTTGCATTG; reverse: TGCACAAAACCGTCTCATCTTC), *GLIPR1* (forward: TCCGATCAGAGGTGAAACCAA; reverse: GGCTTCAGCCGTGTATTATGTG), *COL8A1* (forward: AAAGGGGAAATTGGGCCTATG; reverse: CTGGTTGCCCTGGTAACCC), *USP11* (forward: CATTGAACGCAAGGTCATAGAGC; reverse: AACAGTGTGAGATTTGCCCAA), *GALNT18* (forward: CCAGAGGTGAGCATCGTGTT; reverse: CTCCTTGAGCAGATGTGGGG), *ARHGAP25* (forward: CTGAGAGACGCTTTTGATGCT; reverse: TCTCGGAGGTAGAGCTTTAACA), *HSD17B14* (forward: TAGGGCCACAATCCGAGAGG; reverse: GAGCAGTTCAATGCCCGTG), and *NNAT* (forward: ACTGGGTAGGATTCGCTTTTCG; reverse: ACACCTCACTTCTCGCAATGG).

For RNA-Seq, total RNA samples that passed the quality tests were submitted to the Biopolymers Facility at Harvard Medical School. The KAPA mRNA HyperPrep kit for Illumina sequencing was used. mRNA was pulled down using oligo-dT beads, and the resulting mRNA was converted into cDNA. The resulting cDNA then became a library through adapter ligation and post-PCR cleanup. RNA-Seq raw reads were 100-bp reverse-stranded paired-end reads with 50 million reads per sample. The reads were trimmed for adapters and filtered by sequencing of Phred quality (≥Q15) using fastp (71). The count table was generated by aligning of reads to the human transcriptome (Ensembl version 98) using kallisto (72), and conversion of transcript counts to gene counts using tximport (73). To filter out low-expressed genes, we kept genes that had counts per million of more than 0.5 in at least 4 samples. Counts were normalized by the weighted trimmed mean of M values (74). To detect differential genes, we used limma, an R package to investigate differential expression analyses (75). *P* values were corrected using the Benjamini-Hochberg false discovery rate (FDR). Hierarchical clustering analysis was used to determine differential gene clusters using a variable cut height approach (76).

### Glucose uptake, active Rho pull-down assays, and DNA methylation ELISA.

For the glucose uptake assay, iMyos grown in a 96-well plate were serum-starved (DMEM/F12 plus 0.25% BSA) overnight, washed, and incubated with Krebs-Ringer bicarbonate HEPES (KRBH) buffer (120 mM NaCl, 10 mM NaHCO_3_, 4 mM KH_2_PO_4_, 1 mM MgSO_4_, 1 mM CaCl_2_, 30 mM HEPES) with 5.5 mmol/L glucose for 15 minutes at 37°C. The cells were then stimulated with 100 nM insulin for 30 minutes and then incubated with 100 μM of 2-(*N*-(7-nitrobenz-2-oxa-1,3-diazol-4-yl)amino)-2-deoxyglucose (2-NBDG) dye in KRBH buffer for 1 hour at 37°C. Cells were washed 3 times with PBS, and the fluorescence was recorded using a plate reader.

For the active RhoA pull-down assay, differentiated iMyos were processed and assayed according to the manufacturer’s protocol. The kit components, including the Rho rabbit antibody, was purchased from Cell Signaling (catalog 8820).

For quantification of global DNA methylation, DNA was extracted from the differentiated iMyos, and DNA samples were processed according to the manufacturer’s protocol to measure levels of 5-methylcytosine through an ELISA reaction (P-1030-48, EpigenTek).

### Statistics.

Data analysis was performed using appropriate unpaired or paired 2-tailed Student’s *t* test (version 8.4.3, GraphPad Prism Software), and *P* < 0.05 was considered to be significant.

### Study approval.

The iPSC lines used in this study were generated from 20 human study participants who had been recruited and assessed for insulin sensitivity at the Stanford Clinical and Translational Research Unit at Stanford University (Stanford, CA) as part of the NIH-sponsored GENESiPS project, which had approval to conduct the study (69).

### Data availability.

RNA-Seq raw data were deposited to the Gene Expression Omnibus (GEO) database (accession GSE244307). Raw values for all data points in graphs are reported in the Supporting Data Values file.

## Author contributions

NH designed and performed all the experiments, analyzed all the data, designed the figures, and wrote the paper. CRK conceived the study, helped with data analysis and interpretation, reviewed and edited the manuscript, and supervised the project. Both authors read, reviewed, and edited the manuscript.

## Supplementary Material

Supplemental data

Supplemental table 1

Supplemental table 2

Supplemental table 3

Supporting data values

## Version 1 11/30/2023

In-Press Preview

## Version 2 01/16/2024

Electronic publication
