# Supplementary material for: Interactions among insulin resistance, epigenetics, and donor sex in gene expression regulation of iPSC-derived myoblasts
Source: J Clin Invest. 2024 Jan 16;134(2):e172333. doi: 10.1172/JCI172333 (PMC10786688; doi:10.1172/JCI172333)
Supplement: Supplemental data [file jci-134-172333-s062.pdf]

Supplemental Data Figure 1: Clinical characteristics (from (16))

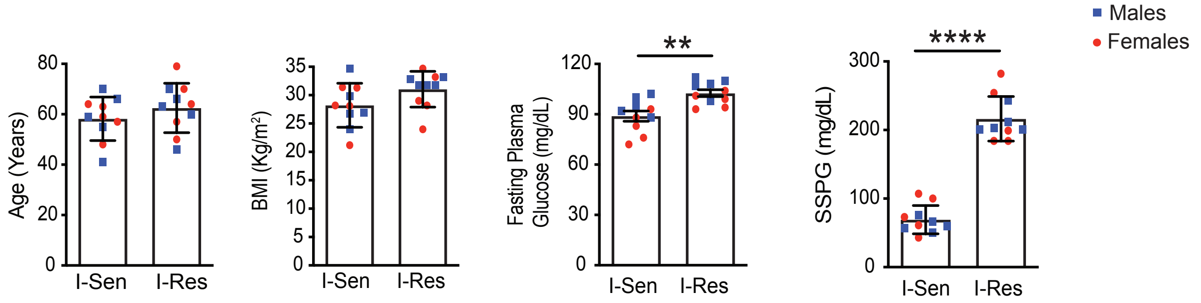

Supplemental Data Figure 2: Biological pathways and genomic distribution of the gene expression changes associated with insulin resistance

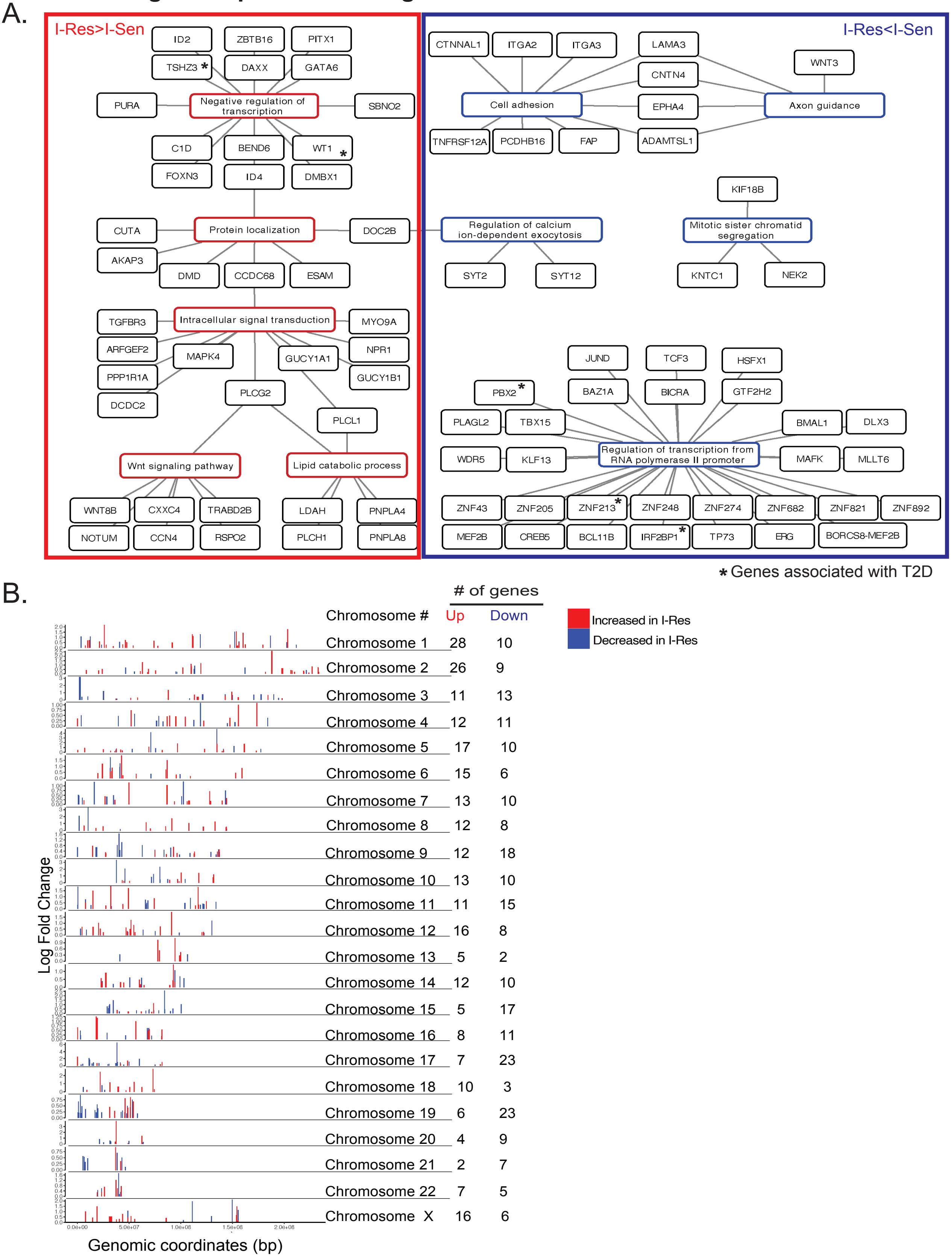

Supplemental Data Figure 3: T2D SNP associations in I-Res iMyos

Gene	Altered in I-Res iMyos	Function and T2D association	Chromosome
SOAT1	Increased	Catalyzes the formation of fatty acid-cholesterol esters, SOAT1 inhibition reduces podocyte injury in diabetic kidney disease [1]	Chr 1
ACP6	Increased	Lysophosphatidic acid phosphatase activity, increased in skeletal muscle biopsies of people with T2D family history [2]	Chr 1
TRIM63	Increased	Muscle E3 ubiquitin ligase, increased in muscle of T1D mice [3]	Chr 1
KCNJ13	Increased	Inward rectifier potassium channel activity, Up in T1D human islets [4]	Chr 2
LSAMP	Increased	Post-translational modification: synthesis of GPI-anchored proteins, levels positively correlates with HOMA-IR [5]	Chr 3
ABHD6	Increased	Enables acylglycerol lipase activity, ABHD6 KO mice are protected from obesity and T2D [6]	Chr 3
FBXW7	Decreased	Ubiquitin protein ligase binding, decreased in muscle of T2D rats [7]	Chr 4
LRRC66	Increased	Cell adhesion, cellular trafficking, and hormone-receptor interactions, altered in rat islets of intrauterine growth retardation, which is associated with development of T2D [8]	Chr 4
ANKDD1B	Decreased	Signal transduction, predicted loss of function SNP in T2D [9]	Chr 5
NADK2	Increased	Mitochondrial kinase that catalyzes yield of NADP, NAD+ precursor treatment is associated with muscle insulin resistance in humans [10]	Chr 5
HLA-B	Decreased	Peptide antigen binding, HLA-B polymorphisms are present in T2D [11]	Chr 6
PBX2	Decreased	DNA-binding transcription factor activity, polymorphism associated with vascular complications in diabetes [12]	Chr 6
LSM2	Decreased	RNA binding and small GTPase binding, altered in T1D patients [13]	Chr 6
SYNGAP1	Increased	Ras GTPase activating protein	Chr 6
RGS17	Increased	GTPase activator activity, associated with diabetes in GWAS [14]	Chr 6
DDAH2	Increased	Regulation of nitric oxide generation, involved in myocardial fibrosis in diabetic cardiomyopathy [15]	Chr 6
STK19	Increased	Protein serine/threonine kinase activity, associated with diabetes in GWAS [14]	Chr 6
HLA-DQA1	Increased	Peptide antigen binding and MHC class II receptor activity, associated with diabetes in GWAS [14]	Chr 6
TRIM26	Increased	E3 ubiquitin-protein ligase which regulates the IFN-beta production, contains T1D associated SNP [16]	Chr 6
TONSL	Decreased	Negative regulator of NF-kappa-B mediated transcription, polymorphisms associated with T2D [17]	Chr 8
GINS4	Decreased	Initiation and progression of DNA replication, associated with β cell dysfunction [18]	Chr 8
ASAH1	Increased	A member of the acid ceramidase family of proteins, associated with T2D in obese population [19]	Chr 8
WT1	Increased	Nucleic acid binding and sequence-specific DNA binding, negative regulator of IGF1 receptor in Wilms tumor biology [20]	Chr 11
RPUSD2	Decreased	Involved in mRNA pseudouridine synthesis	Chr 15
VPS13C	Increased	Mitochondrial function and maintenance, T2D associated gene in β cells [21]	Chr 15
CMIP	Decreased	T cell signaling pathway, polymorphisms associated with T2D [22]	Chr 16
ZNF213	Decreased	DNA-binding transcription factor activity	Chr 16
IRF2BP1	Decreased	Involved in protein polyubiquitination, altered in T2D muscles [23]	Chr 19

TSHZ3	Increased	Transcriptional regulator involved in developmental processes, *Tshz3* haploinsufficiency leads to abnormalities in mouse kidney [24]	Chr 19
PCSK1N	Decreased	Endopeptidase inhibitor activity, involved in proinsulin processing [25]	Chr X
TIMM17B	Increased	Facilitates the transport of mitochondrial proteins, related to diabetic retinopathy [26]	Chr X
KCND1	Increased	Monoatomic ion channel activity, associated with T2D [27]	Chr X

Supplemental Data Figure 4: Autosomal sex-specific gene expression changes are independent of the androgen receptor action

A.

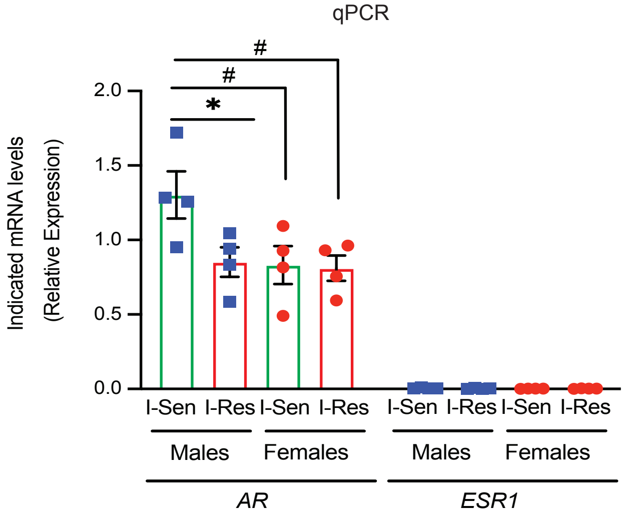

B.

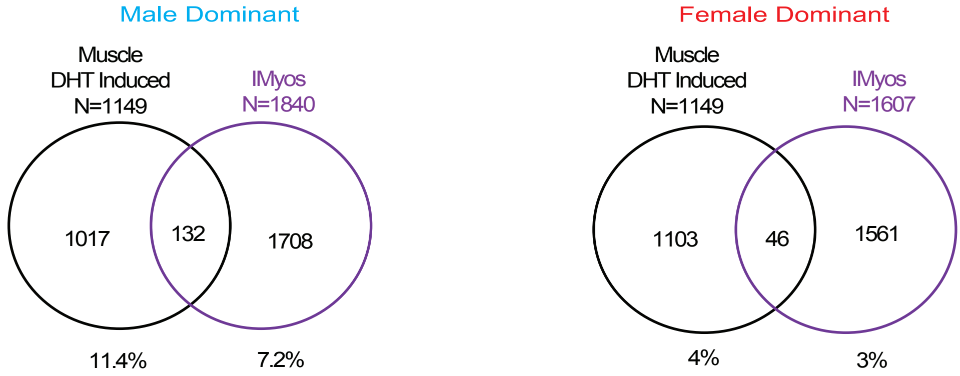

Supplemental Data Figure 5: Sex-specific regulation of epigenetic genes and RhoA isoform and activation

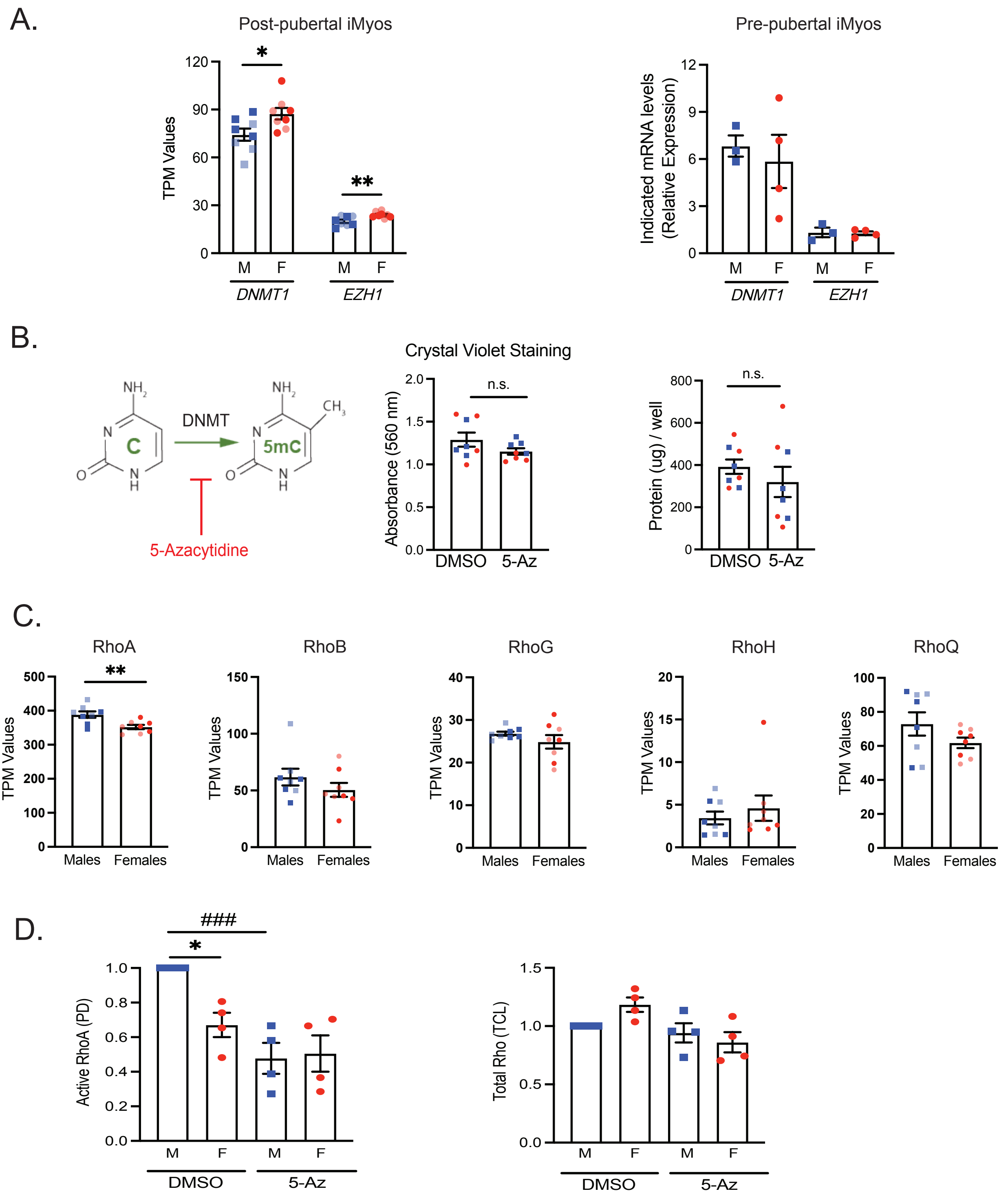

References for Supplemental Data Figure 3

1 Liu, X. *et al.* Sterol-O-acyltransferase-1 has a role in kidney disease associated with diabetes and Alport syndrome. *Kidney Int* **98**, 1275-1285 (2020). https://doi.org:10.1016/j.kint.2020.06.040

2 Elgzyri, T. *et al.* First-degree relatives of type 2 diabetic patients have reduced expression of genes involved in fatty acid metabolism in skeletal muscle. *J Clin Endocrinol Metab* **97**, E1332-1337 (2012). https://doi.org:10.1210/jc.2011-3037

3 O'Neill, B. T. *et al.* FoxO Transcription Factors Are Critical Regulators of Diabetes-Related Muscle Atrophy. *Diabetes* **68**, 556-570 (2019). https://doi.org:10.2337/db18-0416

4 Vastrad, B. V., C. Identification of candidate biomarkers and pathways associated with type 1 diabetes mellitus using bioinformatics analysis *bioRxiv* (2021).

5 Curran, A. M. *et al.* A proteomic signature that reflects pancreatic beta-cell function. *PLoS One* **13**, e0202727 (2018). https://doi.org:10.1371/journal.pone.0202727

6 Zhao, S. *et al.* alpha/beta-Hydrolase Domain 6 Deletion Induces Adipose Browning and Prevents Obesity and Type 2 Diabetes. *Cell Rep* **14**, 2872-2888 (2016). https://doi.org:10.1016/j.celrep.2016.02.076

7 Zhang, W. *et al.* Comprehensive analysis of long non-coding RNAs and mRNAs in skeletal muscle of diabetic Goto-Kakizaki rats during the early stage of type 2 diabetes. *PeerJ* **8**, e8548 (2020). https://doi.org:10.7717/peerj.8548

8 Lien, Y. C., Wang, P. Z., Lu, X. M. & Simmons, R. A. Altered Transcription Factor Binding and Gene Bivalency in Islets of Intrauterine Growth Retarded Rats. *Cells* **9** (2020). https://doi.org:10.3390/cells9061435

9 Vujkovic, M. *et al.* Discovery of 318 new risk loci for type 2 diabetes and related vascular outcomes among 1.4 million participants in a multi-ancestry meta-analysis. *Nat Genet* **52**, 680-691 (2020). https://doi.org:10.1038/s41588-020-0637-y

10 van de Weijer, T. *et al.* Evidence for a direct effect of the NAD+ precursor acipimox on muscle mitochondrial function in humans. *Diabetes* **64**, 1193-1201 (2015). https://doi.org:10.2337/db14-0667

11 Jan, A. *et al.* Association of HLA-B Gene Polymorphisms with Type 2 Diabetes in Pashtun Ethnic Population of Khyber Pakhtunkhwa, Pakistan. *J Diabetes Res* **2021**, 6669731 (2021). https://doi.org:10.1155/2021/6669731

12 Hudson, B. I., Stickland, M. H., Futers, T. S. & Grant, P. J. Effects of novel polymorphisms in the RAGE gene on transcriptional regulation and their association with diabetic retinopathy. *Diabetes* **50**, 1505-1511 (2001). https://doi.org:10.2337/diabetes.50.6.1505

13 Li, C., Wei, B. & Zhao, J. Competing endogenous RNA network analysis explores the key lncRNAs, miRNAs, and mRNAs in type 1 diabetes. *BMC Med Genomics* **14**, 35 (2021). https://doi.org:10.1186/s12920-021-00877-3

14 Li, M. J. *et al.* GWASdb v2: an update database for human genetic variants identified by genome-wide association studies. *Nucleic Acids Res* **44**, D869-876 (2016). https://doi.org:10.1093/nar/gkv1317

15 Zhu, Z. D. *et al.* DDAH2 alleviates myocardial fibrosis in diabetic cardiomyopathy through activation of the DDAH/ADMA/NOS/NO pathway in rats. *Int J Mol Med* **43**, 749-760 (2019). https://doi.org:10.3892/ijmm.2018.4034

16 Nyaga, D. M., Vickers, M. H., Jefferies, C., Perry, J. K. & O'Sullivan, J. M. Type 1 Diabetes Mellitus-Associated Genetic Variants Contribute to Overlapping Immune Regulatory Networks. *Front Genet* **9**, 535 (2018). https://doi.org:10.3389/fgene.2018.00535

17 Klyosova, E., Azarova, I. & Polonikov, A. A Polymorphism in the Gene Encoding Heat Shock Factor 1 (HSF1) Increases the Risk of Type 2 Diabetes: A Pilot Study Supports a Role for Impaired Protein Folding in Disease Pathogenesis. *Life (Basel)* **12** (2022). https://doi.org:10.3390/life12111936

18 Thomsen, S. K. *et al.* Systematic Functional Characterization of Candidate Causal Genes for Type 2 Diabetes Risk Variants. *Diabetes* **65**, 3805-3811 (2016). https://doi.org:10.2337/db16-0361

19 Chathoth, S. *et al.* Insulin resistance induced by de novo pathway-generated C16-ceramide is associated with type 2 diabetes in an obese population. *Lipids Health Dis* **21**, 24 (2022). https://doi.org:10.1186/s12944-022-01634-w

20 Werner, H. *et al.* Increased expression of the insulin-like growth factor I receptor gene, IGF1R, in Wilms tumor is correlated with modulation of IGF1R promoter activity by the WT1 Wilms tumor

gene product. *Proc Natl Acad Sci U S A* **90**, 5828-5832 (1993). https://doi.org:10.1073/pnas.90.12.5828
21 Mehta, Z. B. *et al.* Changes in the expression of the type 2 diabetes-associated gene VPS13C in the beta-cell are associated with glucose intolerance in humans and mice. *Am J Physiol Endocrinol Metab* **311**, E488-507 (2016). https://doi.org:10.1152/ajpendo.00074.2016
22 Cao, Y. *et al.* Opposite Genetic Effects of CMIP Polymorphisms on the Risk of Type 2 Diabetes and Obesity: A Family-Based Study in China. *Int J Mol Sci* **19** (2018). https://doi.org:10.3390/ijms19041011
23 Frederiksen, C. M. *et al.* Transcriptional profiling of myotubes from patients with type 2 diabetes: no evidence for a primary defect in oxidative phosphorylation genes. *Diabetologia* **51**, 2068-2077 (2008). https://doi.org:10.1007/s00125-008-1122-9
24 Sanchez-Martin, I. *et al.* Haploinsufficiency of the mouse Tshz3 gene leads to kidney defects. *Hum Mol Genet* **31**, 1921-1945 (2022). https://doi.org:10.1093/hmg/ddab362
25 Liu, T. *et al.* Pax6 directly down-regulates Pcsk1n expression thereby regulating PC1/3 dependent proinsulin processing. *PLoS One* **7**, e46934 (2012). https://doi.org:10.1371/journal.pone.0046934
26 Zhong, Q. & Kowluru, R. A. Diabetic retinopathy and damage to mitochondrial structure and transport machinery. *Invest Ophthalmol Vis Sci* **52**, 8739-8746 (2011). https://doi.org:10.1167/iovs.11-8045
27 Pletscher-Frankild, S., Palleja, A., Tsafou, K., Binder, J. X. & Jensen, L. J. DISEASES: text mining and data integration of disease-gene associations. *Methods* **74**, 83-89 (2015). https://doi.org:10.1016/j.ymeth.2014.11.020
